# 1691. Use of multiplex PCR in pleural effusion: a case series study in a pediatric reference center in Colombia

**DOI:** 10.1093/ofid/ofad500.1524

**Published:** 2023-11-27

**Authors:** Juan Pablo Londono-Ruiz, Andrea Vargas-Garcia, Adriana Cardenas-Muller, Ana Maria Bejarano-Quintero, Maria Ximena Mantilla, Ivan Felipe Gutierrez-Tobar

**Affiliations:** Clinica Infantil Colsubsidio, Staphylored Colombia, Bogota, Distrito Capital de Bogota, Colombia; Instituto Nacional de Pediatria, Ciudad de Mexico, Distrito Federal, Mexico; Clinica Infantil Colsubsidio, Bogota, Distrito Capital de Bogota, Colombia; Clinica Infantil Colsubsidio, Bogota, Distrito Capital de Bogota, Colombia; Clinica Infantil Colsubsidio, Bogota, Distrito Capital de Bogota, Colombia; Clínica Infantil Santa Maria del Lago y Clinica Infantil Colsubsidio, Bogota, Distrito Capital de Bogota, Colombia

## Abstract

**Background:**

The microbiological diagnosis of pleural effusion (PE) is largely based on methods of classical microbiology. Culture-based methods allow for testing of bacterial susceptibility tests, but these methods take time and frequently have false negative results. We describe the utility of multiplex PCR in pleural fluid for the diagnosis of complicated pneumonia in children using a commercial platform for respiratory samples.

**Figure:**
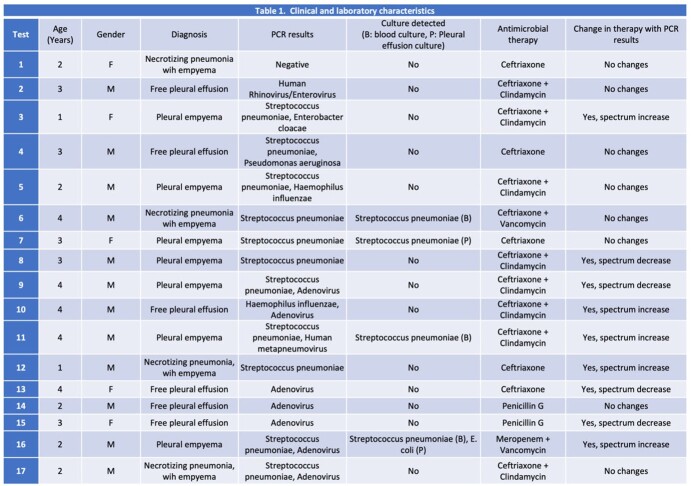

**Methods:**

We conducted a case series of children with complicated pneumonia by PE, empyema or necrotizing pneumonia. Pleural fluid was sent for laboratory to culture, cytochemical and molecular biology testing analysis between 2021 and 2022 in a reference children hospital in Bogotá, Colombia. We used BIOFIRE® FILMARRAY® Pneumonia Panel plus (BioFire Diagnostics, Salt Lake City, Utah). The results were strictly analyzed by a multidisciplinary team. Any change in patient’s therapeutics was decided by this group.

**Results:**

We identified 17 samples that were processed for multiplex-PCR from 16 patients (Table 1). Median age was 3 years (IQR 2-4 years). Pleural empyema was the most common presentation (7/17, 41%), followed by free pleural effusion (6/17, 35%) and necrotizing pneumonia with empyema (4/17, 23,5%). Pathogens were documented in 16 out of 17 (94%) molecular panels performed. *Streptococcus pneumoniae* was detected in 11/17 (64.7%) multiplex PCR tests, adenovirus was detected in 7/17 (41.1%), and *H. influenzae* in 2/17 (11.7%) tests. Pneumococcus was isolated in classical cultures in 4 patients (23.5%), of which only one was in pleural fluid. Changes in antibiotic management in 9/17 (52.9%) of the patients. In 5 patients, there was an increase in the antibiotic spectrum, and in 4 patients, there was a decrease in it.

**Conclusion:**

Although the use of this multiplex PCR device is not approved by the FDA for use in pleural fluid samples (off-label), this case series described its usefulness and importance in contexts where there is no other approved PCR technology for these samples.

**Disclosures:**

**All Authors**: No reported disclosures

